# Interrater agreement of multi-professional case review as reference standard for specialist palliative care need: a mixed-methods study

**DOI:** 10.1186/s12904-023-01281-7

**Published:** 2023-11-16

**Authors:** Evelyn Müller, Michael Josef Müller, Katharina Seibel, Christopher Boehlke, Henning Schäfer, Carsten Klein, Maria Heckel, Steffen T. Simon, Gerhild Becker

**Affiliations:** 1 Department of Palliative Medicine, Medical Center – University of Freiburg, Faculty of Medicine, University of Freiburg, Robert-Koch-Straße 3, 79106 Freiburg, Germany; 2 Department of Palliative Care, University Hospital Basel, Petersgraben 4, 4031 Basel, Switzerland; 3 Department of Radiation Oncology, Medical Center, Faculty of Medicine, University of Freiburg, German Cancer Consortium (DKTK) Partner Site Freiburg, German Cancer Research Center (DKFZ), Heidelberg, Robert-Koch-Straße 3, 79106 Freiburg, Germany; 4 Department of Palliative Medicine, University Hospital Erlangen-EMN, Comprehensive Cancer Center CCC Erlangen, Friedrich-Alexander-University Erlangen-Nürnberg, Krankenhausstraße 12, 91054 Erlangen, Germany; 5 Department of Palliative Medicine and Center for Integrated Oncology Aachen Bonn Cologne Dusseldorf (CIO ABCD), University Hospital of Cologne, Faculty of Medicine and University Hospital, Kerpener Str. 62, 50937 Cologne, Germany

**Keywords:** Referral and consultation, Palliative care, Neoplasms, Psychometrics

## Abstract

**Background:**

A wide variety of screening tools for the need for specialist palliative care (SPC) have been proposed for the use in oncology. However, as there is no established reference standard for SPC need to compare their results with, their sensitivity and specificity have not yet been determined. The aim of the study was to explore whether SPC need assessment by means of multi-professional case review has sufficient interrater agreement to be employed as a reference standard.

**Methods:**

Comprehensive case descriptions were prepared for 20 inpatients with advanced oncologic disease at the University Hospital Freiburg (Germany). All cases were presented to the palliative care teams of three different hospitals in independent, multi-professional case review sessions. The teams assessed whether patients had support needs in nine categories and subsequently concluded SPC need (yes / no). Interrater agreement regarding SPC need was determined by calculating Fleiss’ Kappa.

**Results:**

In 17 out of 20 cases the three teams agreed regarding their appraisal of SPC need (substantial interrater agreement: Fleiss’ Kappa κ = 0.80 (95% CI: 0.55–1.0; *p* < 0.001)). The number of support needs was significantly lower for patients who all teams agreed had no SPC need than for those with agreed SPC need.

**Conclusions:**

The proposed expert case review process shows sufficient reliability to be used as a reference standard. Key elements of the case review process (e.g. clear definition of SPC need, standardized review of the patients’ support needs) and possible modifications to simplify the process are discussed.

**Trial registration:**

German Clinical Trials Register, DRKS00021686, registered 17.12.2020.

**Supplementary Information:**

The online version contains supplementary material available at 10.1186/s12904-023-01281-7.

## Background

The issue of identifying patients in need of specialist palliative care (SPC) in oncology has occupied experts for more than a decade [1–3], and the widespread introduction of screening for SPC need is currently being sought [4, 5]. Accordingly, a wide variety of screening tools have been proposed: Some are generic [6, 7], while others are indication- [1, 3, 8, 9] or entity-specific [10–13]. The majority of the tools require assessment of criteria by staff [1, 3, 8, 9]; some authors suggest the use of patient-reported outcome measures [14, 15]. The proposed screening tools include a wide variety of criteria: disease-, treatment- and care-related aspects, as well as patient and family needs [2, 3, 8, 9, 16]. Although all these tools have very similar objectives, the suggested criteria for determining SPC need differ considerably among them.

In practice, the question arises as to which of the proposed screening tools should be employed. Which assessment best identifies patients with SPC need (sensitivity) and at the same time sorts out those who do not need SPC as reliably as possible (specificity)? Determining sensitivity and specificity requires a reference standard against which the results of the screening tool can be compared. A reference standard is defined as the best available method for determining the presence or absence of the target condition; it differs from a gold standard which is error-free [17]. To our knowledge, no validated reference standard has yet been suggested for determining the need for SPC in oncology patients [16, 18]. In its absence, individual screening instruments have been shown to correlate with various parameters, such as SPC referral [9, 19], mortality / prognosis [2, 6, 16, 19, 20] and patient need questionnaires [19]. But do these parameters accurately and reliably reflect SPC need?

SPC referral and reception are closely related to SPC need. Unfortunately, in current practice, it cannot be assumed that patients reliably receive SPC when they need it. Oncologists’ views, awareness and knowledge of SPC [21–23], resources and patient wishes, for example, can all influence SPC referral [24], making it a very imprecise measure of actual SPC need.

Mortality and prognosis (e.g. surprise question) can be easily measured [25]. They are employed in validation studies of screening tools for SPC need based on the concept of early integration of palliative care (PC) [2, 15]. Early integration is typically defined as referral to palliative care within 2–3 months of diagnosis of advanced disease in cancer patients, as symptom burden increases on average as death approaches [26–29]. Due to limited resources in SPC and data suggesting that SPC mainly benefits patients with pre-identified support needs but not those without support needs [30], the concept of timely integration of SPC was introduced. Timely integration shifts the focus away from disease progression and aims “to identify patients with high support care needs and to refer these individuals to specialist palliative care in a timely manner” [4]. If the aim is to identify patients for timely integration, prognosis and mortality surely correlate with SPC need but are not a suitable sole reference standard.

Patient reported outcome measures like the Integrated Palliative Outcome Scale (IPOS) [31] or Edmonton Symptom Assessment System (ESAS) [32] cover a broad and relevant range of patient needs and thus reflect the idea of needs-based, timely integration well. IPOS has been employed for validation of the screening of criteria proposed by the National Comprehensive Cancer Network (NCCN) [19]. Conversely, both IPOS and ESAS have been proposed as screening tools for SPC need, including initial suggestions for cut-offs [14, 15]. However, to our knowledge, it has never been confirmed that the questionnaires themselves reflect SPC need, and the proposed cut-offs have also not been sufficiently validated.

When there is no gold standard, clinical expertise is frequently used as a reference standard [33]. We are aware of two recently published studies that employed clinical expertise as the reference standard for SPC need assessment: Teike Lüthi et al. (2021) asked a PC team of physician and nurse to determine SPC need [34], Effendy et al. (2022) used independent assessments made by the treating oncology physician and nurse [35]. Neither of the studies report interrater agreement of the reference standard.

The reported study is a pre-study to validate the reference standard in the context of the ScreeningPall study, which aims to develop easy-to-use screening criteria for SPC need (study protocol see [36]).

The aim of this pre-study was to explore whether SPC need assessment by means of multi-professional case review has sufficient interrater agreement to be employed as a reference standard. Additionally, we assessed the challenges in its use as a reference standard for SPC need.

## Methods

### Study design

The pre-study was designed as a mixed-methods study, data were collected between 08/2021 and 11/2022. It combines a reliability study, in which three PC teams independently assessed the same 20 patients with metastatic or locally advanced incurable cancer in structured multi-professional case reviews regarding SPC need, with a qualitative analysis of case review transcripts to identify challenges.

### Definition of SPC need

In our study, we employ a definition of SPC need that is aligned with the requirements of the German health system. The question in case review is (translated from German): ‘Due to the needs of the patient and the relatives, is there currently a challenging situation with complex symptoms (physical, psychological, social or spiritual) that requires specialist palliative care? Specialist palliative care is characterized by practitioners with specific palliative care qualifications and experience, a multi-professional team approach and 24 h availability’ (based on the German S3-Guideline [13]). The teams were asked to focus on the current presence of SPC need and to assess it independently of the service that would carry out the SPC (e.g. consultation service, outpatient service). There also was a lower-level option of an ‘advisory session to inform the patient about possibilities and accessibility of specialised palliative care’, which could be chosen e.g. in cases of possible future need. It did not count as ‘SPC need’.

### Setting

The pre-study was conducted at the University Medical Center Freiburg in Germany, a tertiary care centre with a Comprehensive Cancer Center. Patients were recruited in inpatient radiotherapy. The two cooperating PC teams from Erlangen and Cologne are also located at large university hospitals with Comprehensive Cancer Centers in Germany.

### Patient sample

Inclusion criteria for patients were metastatic or locally advanced, incurable cancer with low probability of long-term control of the disease (estimated survival < 2 years) as assessed by the treating physician, ≥ 18 years of age and informed consent by patient or proxy. Patients with malignant haematological diseases as their main oncological diagnosis were excluded. We aimed to include 20 case descriptions for case reviews with approximately equal numbers of patients with and without SPC need for determination of interrater agreement. The sample size was based on a pragmatic decision, 20 cases was the maximum number for which the effort was still considered feasible for the external teams. Patient recruitment was to be terminated when 10 patients had been assessed as having SPC need by the Freiburg team. Due to recruitment of a higher number of patients without SPC need than with SPC need, we selected patients without SPC need with the aim of obtaining a heterogeneous sample regarding tumour entity, age and gender.

Case descriptions were based on information from routine documentation and standardized medical history taking by a physician and / or nurse from the PC team during a bedside visit (for details see Additional file 1).

### Multi-professional case review

**Fig. 1:**
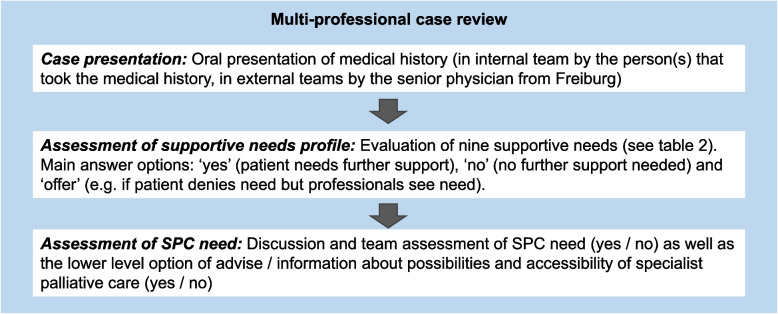
Overview of case review process

Each case was discussed by the three PC teams from Freiburg (internal team), Erlangen and Cologne university hospitals (external teams), without them knowing the results from the other PC teams. PC teams consisted of at least one physician, one nurse, one social worker or case manager, and one psychologist or pastoral worker (four different professional groups). An overview of the structured process of the multi-professional case review is presented in Fig. 1. The case reviews were digitally recorded and transcribed verbatim; the outcome and observations on the process were documented in writing on site (for further information see Additional file 1).

### Statistical and qualitative analysis

Fleiss' Kappa was calculated to test the interrater agreement on SPC need among the three teams [37], employing IBM SPSS 28 Software [38]. Descriptive statistics and the median test (alpha = 5%, two-sided) were used for an exploratory analysis of differences in support needs profiles among the three case groups ‘agreement SPC need’, ‘agreement no SPC need’ and ‘no agreement regarding SPC need’. There were no missing values.

For the cases in which the three teams did not agree regarding SPC need, a qualitative analysis [39] was conducted to identify possible factors that caused the differing conclusions. A team of four (social worker, social scientist, psychologist, nurse/medical documentarist; two members of the study team, two not involved) first read the transcripts line by line individually and annotated them, taking into account pragmatic, syntactic and semantic aspects. The team then developed a code system for possible influencing factors. This consisted of the nine predefined support needs categories and the SPC needs category (see Additional file 1), as well as the possible influencing factors identified in the team discussion: missing/additional information in the presentation of the patient cases, interruptions and interactions in the case presentation, and the roles of case presenter and facilitators (local senior physicians). Subsequently, the transcripts were coded, i.e. the texts were examined sequentially and the text passages were assigned to categories and discussed in the team. The results of the team discussions were recorded in case summaries and these were finally compared in a cross-evaluation with regard to the similarities and differences of the influencing factors.

## Results

### Patient sample

History taking and case review took place for 32 patients before we reached 10 patients for whom the Freiburg team had concluded SPC need (criterion for recruitment termination). Two cases were excluded due to problems in medical history taking (e.g. focus on emotional support instead of medical history taking). Of the 30 remaining cases 20 were selected (for characteristics see Table 1): Of the 30 cases nine were assessed as having SPC need, 21 as having no SPC need by the internal team. All patients with SPC need were included with the aim of a well-balanced ratio of cases with and without SPC need. Of the 21 patients assessed as having no SPC need by the interal team, two were included due to special interest after controversial discussion in the internal team and nine based on the pre-defined criterion of heterogeneity regarding tumour entity, gender and age. There were no missing data on the relevant variables for the selected cases (for characteristics of excluded/non-selected cases, see Additional file 2).

**Table 1:** Characteristics of the patient sample (*n* = 20)

	**SPC need as initially assessed by Freiburg PCT**		**Total sample**
	**Yes** (*n* = 9)	**No** (*n* = 11)	
**Gender**			
Female	4	4	8
Male	5	7	12
**Age** (years)			
≤ 35	0	1	1
36–50	0	2	2
51–65	5	4	9
66–80	3	3	6
> 80	1	1	2
**Main diagnosis.** Malignant neoplasms of…			
C00-C14: lip, oral cavity and pharynx	1	0	1
C15-C26: digestive organs	1	4	5
C30-C39: respiratory and intrathoracic organs	4	2	6
C43-C44: melanoma / skin	0	2	2
C51-C58: female genital organs	1	1	2
C60-C63: male genital organs	1	0	1
C64-C68: urinary tract	1	1	2
C69-C72: eye, brain, central nervous system	0	1	1

### PC teams

Between four and nine professionals took part in case review sessions, with variations among teams. The composition of the Freiburg team changed weekly according to the duty rosters; in the external teams, there were two block appointments for 10 case reviews each and team compositions were consistent. In all case reviews, at least four different professional groups were represented.

### Interrater agreement and assessment of SPC need in the three PC teams

In 17 of the 20 cases, there was agreement among the three PC teams on whether the patients currently had a need for SPC. This corresponds to an interrater agreement of Fleiss’ Kappa κ = 0.80 (95% CI: 0.55–1.0; *p* < 0.001), which indicates substantial agreement [37]. The internal team identified SPC need in nine cases, the two external teams in ten and twelve. The agreement for individual cases is shown in Table 2.

**Table 2:**
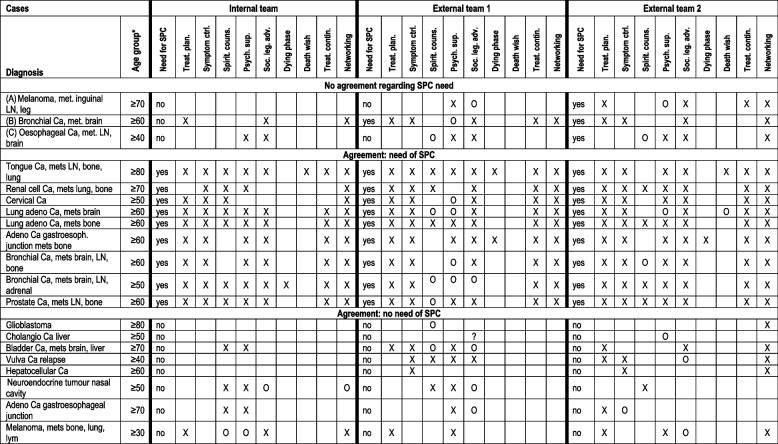
SPC need and support needs profiles comparing the three PCTs

*NEEDS PROFILE:* X yes, O offer

*NEEDS CATEGORY Treat* Plan treatment decision/planning, *Symptom ctrl* Symptom control, *Spirit couns* Spiritual counselling*, Psych. sup* Psychological support, *Soc. leg. adv*. Social legal advice, *Dying phase* Support in the dying phase, *Treat. contin*. Ensuring treatment continuity, *Networking Information*, networking and coordination of involved practitioners

^*^age within a 10 year range of the stated number

### Support needs profiles and SPC need

Table 2 shows the support needs profiles. Explorative comparison of the needs profiles showed that patients for whom SPC need was identified by all three PC teams had a significantly higher number of support needs in their needs profiles than patients for whom SPC need was consistently not identified (see Table 3). In the three cases without agreement, the median number of support needs was higher than that of patients without SPC need and lower than that of those with SPC need (due to the small sample size, the median test was not informative).

**Table 3:** Descriptive statistics on number of needs in needs profile depending on SPC need and agreement regarding SPC need among teams

	**Number of ‘yes’ or ‘offer’ answers in needs profile (median (min–max))**			**Result Median-Test**^a^
	Agreement no SPC (*n* = 8)	No agreement (*n* = 3)	Agreement SPC (*n* = 9)	
Internal team	1 (0–5)	2 (0–3)	7 (4–8)	Χ^2^ (2) = 9.9; *p* ≤ .01
External team 1	2 (0–5)	3 (2–6)	7 (6–8)	Χ^2^ (2) = 17.3; *p* ≤ .01
External team 2	1.5 (1–4)	4 (4–5)	7 (6–7)	Χ^2^ (2) = 17.3; *p* ≤ .01

^a^Reported results are results of the global test, if there was any significant difference among the three groups. In post-hoc-tests with bilateral comparisons, only the difference between the groups ‘Agreement no SPC’ and ‘Agreement SPC’ was significant, with comparisons to the group ‘No agreement’ being uninformative due to the small number of cases (*n* = 3)

### Qualitative analysis of cases with no agreement on SPC need

**Fig. 2:**
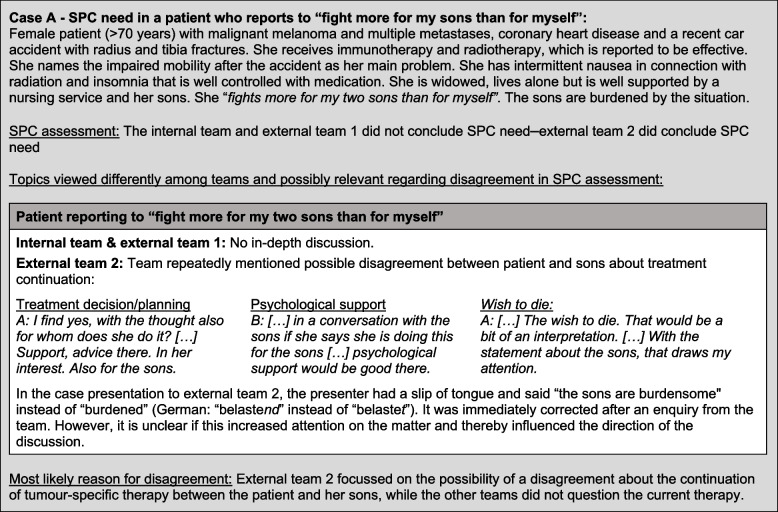
Qualitative analysis of Case A regarding decision making in case reviews on SPC need

Relevant different conclusions of the teams occurred in situations involving (Case A) the *ambiguous patient statement* “I mainly fight for my sons” by a widow with a currently effective therapy regime (see Fig. 2), (Case B) divergent assessment of whether the *family and professional support system in place is capable of handling the situation* of a man with a learning disability who cannot return home to live by himself and (Case C) *anticipation of possible SPC need in the near future* in a man currently with no relevant physical needs but a high need for psycho-spiritual and social work support (Cases B and C in Additional file 3). In Case A, a procedural aspect—a slip of the tongue during the case presentation—might have led to different levels of attention being paid to specific information. Additional file 4 summarises further observations on the case review procedure.

## Discussion

We aimed to explore the use of the clinical expertise of multi-professional teams as a reference standard for SPC need. The substantial agreement among the three teams indicates reliability [37]; however, the confidence interval is still large due to the rather small sample. Further studies might contribute to a more precise estimation of interrater agreement.

The number of needs in the needs profile reflects the concept of timely integration of palliative care [4], where an increasing number of support needs in the course of the illness is associated with the timing of SPC integration. While cases with a high or low number of support needs are easy to agree upon regarding SPC need, the PC teams disagreed about three cases. Examples of case characteristics that led to patients being put into this group are unclear coping skills of the patient and/or support system, ambiguous statements of the patient and possible needs in the near future. For the reference standard, these are sources of error which are difficult to avoid.

Regarding the processes of SPC assessment, the following features should be considered for further development and use as a reference standard:

*How should “SPC need” be defined in studies*? We assumed that a definition of SPC need that is clear and as close to everyday care as possible will be easier to assess and most useful for the subsequent validation of screening tools in our context. Therefore, our definition reflects what we would like to identify: patients who need the expertise and resources of SPC due to high support needs [13].

*What information should the reference standard for SPC need be based on?* Patients’ SPC need is determined by their current support needs, and whether they and their support system can cope [40]. In their study, Teike Lüthi et al. based their assessment on computer-based patient records alone or on patient records combined with palliative care liaison rounds [34]. The appropriateness of that approach depends on local completeness and the timelines of information in the records. In our study, medical history taking was necessary, as patient records did not contain sufficient information e.g. on the coping of patients and the support system.

*Who should make the assessment of the reference standard for SPC need?* Considering studies that show the impact of professionals' knowledge, experience and sensitisation in palliative care on the assessment of SPC need [21–23], we believe that experienced palliative care professionals should carry out the assessment as opposed to treating professionals. The approach of Teike Lüthi et al. involving assessment by dyads [34] instead of multi-professional teams is promising, especially combined with a holistic needs profile. As physicians and nurses in palliative care are usually attentive and sensitive to psycho-spiritual needs [41, 42], the participation of psychologists and social workers might not be necessary.

### Strengths of the study

To our knowledge our study is the first to show sufficient interrater agreement of expert opinion on SPC need and explore the prerequisites of the assessment processes. The suggested approach is not a gold standard but a reference standard, as the assessment cannot be free of error [17]. However, just like the surprise question for determining prognosis, it might be the best available option for the complex assessment of SPC need. The suggested procedure is transferable to other settings and healthcare systems, applicable and likely reliable.

### Limitations of the study

Our results are based on data from only 20 patients. A larger patient sample would allow a more precise measurement of interrater agreement and further exploration of patient case characteristics and process features that might cause difficulties in the assessment of SPC need.

Case reviews were not strictly blinded as the senior physician at Freiburg presented the cases to the external expert teams. The background to that decision was a pre-test with Freiburg staff not involved in the study, in which the written case descriptions were prepared in advance (see Additional file 1). It became apparent that the high effort of working through the 20 case descriptions resulted in selective attention to certain information based on personal / professional priorities and in a game of predicting the team's answers in terms of SPC needs (and thus pre-judgment). Consequently, we preferred oral case presentation by a Freiburg team member, allowing for the exact same procedure as in Freiburg and the presence of a person with further in-depth information if needed. Guidelines for the senior physician stipulated that he would only speak during team review and assessment phases if asked for additional information on the case. In reality, however, non-verbal reactions cannot be fully ruled out and transcripts reveal minor deviations from the guideline to not speak, e.g. repeated discussion in one external team about the differences in radiotherapy use between the two hospitals, triggered by questions to the case presenter from the external senior physician.

Case selection was not random and the predefined rule of heterogeneity was not followed as strictly as it could have been. For example, the case C (no current physical but high psychological needs) was deliberately included after controversial discussion in the internal team—and the three teams did not agree on it. The use of real patient cases instead of constructed, controlled cases makes it possible to analyse the challenges of case reviews as reference standard. However, we cannot determine the influence of that selection on the interrater agreement.

The qualitative analysis is based only on the three cases where there was disagreement, as these contribute most to the understanding of pitfalls and the need for adaptation of our processes in future research.

## Conclusions

The approach of medical history taking and structured, multi-professional case review shows sufficient interrater agreement to be employed as a reference standard for SPC needs in studies validating screening tools. However, further research is needed to confirm these results. For use in studies in other countries or care services, we recommend a review and possibly adaption to national and local requirements.

### Supplementary Information

**Additional file 1. **Development and Documentation of the Reference Standard. Detailed description of a process and documentation of medical history taking and case review as reference standard, including final versions of the used documents.

**Additional file 2.** Characteristics of not selected patients. Characteristics of patients not selected for case reviews (*n*=10) and excluded patients (*n*=2).

**Additional file 3.** Additional results of qualitative analysis. Additional results of qualitative analysis of cases with no agreement regarding SPC need assessment.

**Additional file 4.** Observations on the case review procedure. Observations on the case review process, including effort of case reviewers, framework conditions, supportive needs profile, and oral case presentation.

## Data Availability

The datasets generated and/or analysed during the current study are not publicly available due to ensuring data protection and anonymity for the patients, but are available from the corresponding author on reasonable request.
