# Supplementary material for: Interrater agreement of multi-professional case review as reference standard for specialist palliative care need: a mixed-methods study
Source: BMC Palliat Care. 2023 Nov 16;22:181. doi: 10.1186/s12904-023-01281-7 (PMC10652431; doi:10.1186/s12904-023-01281-7)
Supplement: Supplementary file 1 — Additional file 1. Development and Documentation of the Reference Standard. Detailed description of a process and documentation of medical history taking and case review as reference standard, including final versions of the used documents. [file 12904_2023_1281_MOESM1_ESM.docx]

**Additional file 1**

[Development of a process and documentation of medical history taking and case review as reference standard: 1](#_Toc125369435)

[Medical history form for interview with patients / relatives 2](#_Toc125369436)

[Documentation of case discussion 6](#_Toc125369437)

# Methods of Development of a process and documentation of medical history taking and case review as reference standard:

Standard documents were developed do guide the processes of medical history taking and case review. The aim was to ensure standard procedures, e.g. questions to the patient in medical history taking and discussion in case review. For medical history taking the standard everyday medical history taking documents of the PC team in Freiburg were employed as first version. For multi-professional case review the study team suggested a first version of the process and documentation based on literature reporting supportive needs of palliative care patients (1, 2).

Both documents were pretested and explored in individual cognitive interviews with two physicians, three nurses, one social worker, one psychologist and one pastor (PC team Freiburg), to document individual understanding of terms, judgements on relevance of content and missing content in the light of the study question of SPC need in oncologic patients.

In a joint session, differences were discussed and the whole PC team consented a pre-final version; minor adjustments were made after pre-tests in practice. Final versions are attached below. Final procedures are described in the main text of the publication.

Furthermore, for external case review we pre-tested the use of written case descriptions in external teams. A Freiburg physician, who was not in any way involved in the study, was informed about the project aims and asked to elaborate the 20 cases in preparation of a hypothetical team case review. We informally talked about her experience and pros of cons of the use of written vs. oral presentation of cases. It lead to the preference of oral presentation (see limitations in discussion of main text).

| Patient label  (or name and date of birth) |
| --- |

ScreeningPALL:

# *Final Version of:*

# Medical history form for interview with patients / relatives

Date: ______________________

| **Main problems**  What are the things that bother or stress you the most right now?  We are interested in burdens in the physical, mental, social and spiritual domains. |
| --- |
|  |

| **Pain** | |
| --- | --- |
| **Current intensity** (NRS 0-10) | at rest: on exertion: |
| **Amplified by** |  |
| **Relieved by** |  |
| **Duration** | 🞏 continuous 🞏 intermittent |
| **Neuropath. component** | 🞏 yes 🞏 no |

| **Respiration** | | | |
| --- | --- | --- | --- |
| **Dyspnea** at rest | 🞏 none 🞏 mild 🞏 moderate 🞏 severe | **Dyspnea** on exertion | 🞏 none 🞏 mild 🞏 moderate 🞏 severe |
| **Distressing cough** | 🞏 yes 🞏 no | **Oxygen** | 🞏 yes 🞏 no |

| **Gastrointestinal symptoms** | | | |
| --- | --- | --- | --- |
| **Nausea** | 🞏 yes 🞏 no  🞏 temporary 🞏 permanent  🞏 therapy-resistant | **Vomiting** | 🞏 yes 🞏 no  How often: __________ times a day |
| **Loss of appetite** | 🞏 yes 🞏 no | **Weight loss** | 🞏 yes 🞏 no  How much? ____ kg in __ months |
| **Dysphagia** | 🞏 yes 🞏 no | **Xerostomia** | 🞏 yes 🞏 no |
| **Constipation** | 🞏 yes 🞏 no  Last bowel movement: ______________ | **Incontinence** | 🞏 yes 🞏 no  🞏 urine 🞏 stool |

| **Neurological / psychological symptoms** | | | | |
| --- | --- | --- | --- | --- |
| **Vertigo / tendency to fall** | 🞏 yes 🞏 no | | **Sleep disorder** | 🞏 yes 🞏 no |
| **Sorrow / sadness** | 🞏 yes 🞏 no | | **Inner unrest** | 🞏 yes 🞏 no |
| **Relatives** | | | | |
| **Family situation**  Do you have family? Who belongs to the family? | | 🞏 spouse / partner 🞏 children 🞏 parents  🞏 living alone  🞏 other: _______________________________________________ | | |
| **Main caregiver**  Who do you turn to when you are unwell?  Who supports you? | | 🞏 spouse / partner 🞏 children 🞏 parents  🞏 other: _______________________________________________ | | |
| **Availability of main caregiver**  Is the person there when you need help?  Does the person take care of you at home? | |  | | |
| **Occupation of the patient** | |  | | |
| **Burden of the relatives** | | overburdened 🞏 yes 🞏 no  *Notes:* | | |

| **Nursing & care** | |
| --- | --- |
| **Need for assistance with activities of daily living** (e.g. shopping, personal hygiene)  How do you manage your everyday life?  What do you need assistance with in everyday life? | 🞏 none 🞏 slight 🞏 medium 🞏 strong  *Notes:* |
| **Nursing problems due to wounds / decubitus / drainage**  Do you have any wounds or bedsores?  *If so:* How do you care for them? Do you manage them at home? | 🞏 yes 🞏 no  *Notes:* |
| **Level of care** | Level of care:______________ 🞏 none 🞏 applied for |
| **Previous care / aids:** | 🞏 outpatient care 🞏 hospice aides 🞏 SPC  🞏 other: ___________________________________________  🞏 aids: |

| **Psychosocial situation** | | | |
| --- | --- | --- | --- |
| **Understanding of disease** (focus: palliative / curative)  What do you know about your disease?  Is there anything else you would like to know about your disease? |  | | |
| **Difficulty in coping with illness**  (ability to cope with the changed situation (illness))  How do you cope with your illness?  What helps you to cope with your situation? |  | | |
| **Need for support in finding therapy goals**  Do you need more information around (further) therapy?  What do you expect from your treatment? |  | | |
| **Practical problems**  What about burdens in the following domains? *Then name the domains.*  *Other:* Are there any other things that specifically burden you? | 🞏 children to be cared for 🞏 financial concerns  🞏 problems at work 🞏 problems with the living situation  other: | |  |
| **Advance directive / healthcare proxy** | | 🞏 advance directive 🞏 healthcare proxy  🞏 none 🞏 unknown | |
| **Support needs – psychological / spiritual** | | | |
| **Supportive conversations e.g. with psychologist or pastor desired?** | 🞏 yes 🞏 no | | |
| *If yes:* **with whom** | 🞏 psychologist 🞏 pastor  *other:* | | |

| **Spiritual assessment** | |
| --- | --- |
| *If yes above:* **denomination** |  |
| **Is spirituality or religion a resource?**  What gives you strength? What gives you support? |  |
| *If conspicuous in conversation, inquire if necessary:*  **Does the patient report existential suffering, doubts, struggles with faith, or similar?**  **Death wish?** Would you sometimes wish it were all over? |  |

**Observation**

| **Performance status (ECOG)** |
| --- |
| 🞏 0 Fully active, able to carry on all pre-disease performance without restriction  🞏 1 Restricted in physically strenuous activity but ambulatory and able to carry out work of a light or sedentary nature, e.g., light house work, office work  🞏 2 Ambulatory and capable of all selfcare but unable to carry out any work activities; up and about more than 50% of waking hours  🞏 3 Capable of only limited selfcare; confined to bed or chair more than 50% of waking hours  🞏 4 Completely disabled; cannot carry on any selfcare; totally confined to bed or chair |

| **Vegatative anamnesis** | |
| --- | --- |
| **Weakness** | 🞏 yes 🞏 no |

| **Nutritional status** |
| --- |
| 🞏 obese 🞏 normotrophic 🞏 cachectic |

| **Neurological / psychological symptoms** | |
| --- | --- |
| **Reduced vigilance** | 🞏 yes 🞏 no |
| **Disorientation / confusion** | 🞏 yes 🞏 no |
| **Anxiety / panic** | 🞏 yes 🞏 no |

| **Nursing & care** | |
| --- | --- |
| **Problems with the organization of care**  What happens after hospitalization? Do you already know what the next steps are? | 🞏 yes 🞏 no  *Notes:* |
| Please fill in after history-taking: | |
| **Could the survey be conducted in a regular manner?** | 🞏 yes 🞏 no |
| **If no, what were the limitations?** |  |
| **Who provided the information?** | 🞏 patient 🞏 relatives  🞏 ward physician 🞏 nurse  *Notes:* |
| **Who took the medical history?** | 🞏 physician 🞏 nurse |
| **Was or will the ward physician be contacted directly due to an acute need for action?** | 🞏 yes 🞏 no |

# Documentation of case discussion

Patient label

| **Date** | **Participating professions** |
| --- | --- |
|  | 🞎 physician 🞎 nurse  🞎 social work 🞎 pastoral care  🞎 psychology |

| **Burdens with need for action or treatment: yes / no:** | | | | | | | | | | | | |
| --- | --- | --- | --- | --- | --- | --- | --- | --- | --- | --- | --- | --- |
| **Treatment decision/planning** | | | | | **Symptom control** | | | | | **Spiritual counselling** | | |
| - yes | - offer | | - no | | - yes | | | - no | | - yes | - offer | - no |
| - needs covered - insufficient information for yes / no | | | | | - needs covered - insufficient information for yes / no | | | | | - needs covered - insufficient information for yes / no | | |
| - counselling / orientation on disease and treatment of patient and relatives - therapy goal change - advance care planning - emergency plans - assistance with creating advance healthcare directive or healthcare proxy   other: | | | | | - pharmacotherapeutic measures - interventional measures - emergency plans - counselling / guidance of relatives - counselling / guidance of treatment team - nursing measures (e.g. complex wound care)   other: | | | | | - patient-reported need - practitioner-reported need | | |
|  |  |  |  |  |  |  |  |  |  | - questions of meaning / life review - questions of guilt - existential suffering - religious needs - arrangement of farewell and mourning process   other: | | |
| **Psychological support** | | | | | **Social legal advice** | | | | | **Support in the dying phase** | | |
| - yes | - offer | - no | | | - yes | | - offer | | - no | - yes | - no | |
| - needs covered - insufficient information for yes / no | | | | | - needs covered - insufficient information for yes / no | | | | | - needs covered - insufficient information for yes / no | | |
| - patient-reported need - practitioner-reported need | | | | | - organization of further medical / nursing / home care - facilitation of specialist palliative care / hospice care - advice on support options and claims - advance care planning - referral to outpatient hospice service (volunteers) - sorting out financial issues   other: | | | | | comment: | | |
| - coping - support / coping for relatives - support in case of conflicts - biography work - relaxation techniques   other: | | | | |  |  |  |  |  |  |  |  |
| **Wish to die** | | | | | **Ensuring treatment continuity** | | | | | **Information, networking and coordination of involved practitioners** (general practitioners, outpatient SPC, clinicians etc.) | | |
| - yes | - offer | | | - no | - yes | - no | | | | - yes | - no | |
| - needs covered - insufficient information for yes / no | | | | | - needs covered - insufficient information for yes / no | | | | | - needs covered - insufficient information for yes / no | | |
| comment: | | | | | comment: | | | | | comment: | | |

**Need of palliative care**

| **Due to the needs of the patient and the relatives, is there currently a challenging situation with complex symptoms (physical, psychological, social or spiritual) that requires specialist palliative care?**   - *Comment:* **Specialist palliative care** is characterized by specific palliative care qualifications and experience of practitioners, a multi-professional team approach and 24h availability. | | | | | |
| --- | --- | --- | --- | --- | --- |
| - yes | | - no | | | |
| **How „clear “ is the decision for / against SPC need** | | | | | |
|  | 🞎  clear  (no „close decision“) | | | 🞎  not clear  (= very „close decision“) |  |
|  |  |  |  |  |  |
| **Please explain with keyword(s):** | | | | | |
|  | | | | | |
| **IF NO: In your opinion, would a counselling session be helpful for the patient and his/her family to learn about options and accessibility of specialized palliative care (in the sense of timely / early Integration)?**  *This does not specify who will provide this information (PMD, station staff, etc.).* | | | | | |
| - yes | | - no |  | | |
| comment: | | | | | |

**Prognosis**

| **Would you be surprised if this patient died in the next 12 months?** | |
| --- | --- |
| - no, I would not be surprised | - yes, I would be surprised |
| **Would you be surprised if this patient is still alive in 12 months?** | |
| - No, I would not be surprised | - yes, I would be surprised |

# References

1. Temel JS, Greer JA, El-Jawahri A, Pirl WF, Park ER, Jackson VA et al. Effects of Early Integrated Palliative Care in Patients With Lung and GI Cancer: A Randomized Clinical Trial. J Clin Oncol 2017; 35(8):834–41.

2. Bandeali S, Des Ordons AR, Sinnarajah A. Comparing the physical, psychological, social, and spiritual needs of patients with non-cancer and cancer diagnoses in a tertiary palliative care setting. Palliat Support Care 2020; 18(5):513–8.
