# Supplementary material for: Interrater agreement of multi-professional case review as reference standard for specialist palliative care need: a mixed-methods study
Source: BMC Palliat Care. 2023 Nov 16;22:181. doi: 10.1186/s12904-023-01281-7 (PMC10652431; doi:10.1186/s12904-023-01281-7)
Supplement: Supplementary file 2 — Additional file 2. Characteristics of not selected patients. Characteristics of patients not selected for case reviews (n=10) and excluded patients (n=2). [file 12904_2023_1281_MOESM2_ESM.docx]

**Additional file 2**

**Selection of patient cases for inclusion in reliability study:** Medical histories were taken for 32 patients, at that point ten patients had been assessed as having SPC need and recruiting for phase 1 was stopped. Two cases were excluded due to unreliable medical history taking (n=1 high level of emotional distress; n=1 inconclusive statements), one of them with, one of them without SPC need (according to Freiburg case review result).

The nine remaining patients with SPC need were included without further selection. Of the 21 eligible patients without SPC need 11 were selected with the aim of a heterogeneous sample (diagnosis, age, social situation etc.) by a team of one physician, study assistant and research assistant. One case was specifically included due to a very controversial discussion in Freiburg PC team and special interest in the results of other PC teams.

**Table 1: Characteristics of patients not selected for case reviews (n=10) and excluded patients (n=2)**

|  | **Not selected patients**  (no SPC need n=10) | **Excluded patients** (n=2; 1 SPC need, 1 no SPC need) | **All patients** (including selected patients; n=32) |
| --- | --- | --- | --- |
|  |  |  |  |
| **Gender** |  |  |  |
| Female | 4 | 1 | 13 |
| Male | 6 | 1 | 19 |
| **Age group** (years) |  |  |  |
| ≤ 35 | 0 | 1 | 2 |
| 36-50 | 2 | 1 | 5 |
| 51-65 | 5 | 0 | 14 |
| 66-80 | 1 | 0 | 7 |
| > 80 | 2 | 0 | 4 |
| **Main diagnosis.** Malignant neoplasms of… |  |  |  |
| C00-C14: lip, oral cavity and pharynx | 2 | 0 | 3 |
| C15-C26: digestive organs | 1 | 0 | 6 |
| C30-C39: respiratory and intrathoracic organs | 2 | 0 | 8 |
| C43-C44: Melanoma / skin | 1 | 0 | 3 |
| C50-C50: breast | 0 | 1 | 1 |
| C51-C58: female genital organs | 0 | 0 | 2 |
| C60-C63: male genital organs | 0 | 0 | 1 |
| C64-C68: urinary tract | 0 | 0 | 2 |
| C69-C72: eye, brain, central nervous system | 3 | 0 | 4 |
| C73-C75 thyroid and other endocrine glands | 0 | 1 | 1 |
| C76-C80 ill-defined, secondary and unspecified sites | 1 | 0 | 1 |
