# Supplementary material for: Interrater agreement of multi-professional case review as reference standard for specialist palliative care need: a mixed-methods study
Source: BMC Palliat Care. 2023 Nov 16;22:181. doi: 10.1186/s12904-023-01281-7 (PMC10652431; doi:10.1186/s12904-023-01281-7)
Supplement: Supplementary file 3 — Additional file 3. Additional results of qualitative analysis. Additional results of qualitative analysis of cases with no agreement regarding SPC need assessment. [file 12904_2023_1281_MOESM3_ESM.docx]

**Additional file 3**

**Results of qualitative analysis of cases with no agreement regarding SPC need assessment**

| **Case B - SPC need in a patient with learning disabilities:** Male patient with small cell lung carcinoma with cerebral metastasis (> 60 years, IQ of 50, retired from job in luxury car maintenance). The sister (legal guardian) and patient initially decided against tumour-specific therapy. He is currently hospitalised after an emergency reduced vigilance with a tendency to fall. A hydrocephalic impoundment was successfully treated with a shunt, and an inpatient radiotherapy is currently being conducted. He reports feeling “imprisoned” in inpatient treatment as his main burden. He lives by himself in a flat on the fourth floor, supported by his sister and everyday assistance from a welfare service. Due to progression of the illness, he cannot return to live by himself. He greatly enjoys being the mascot of a sports team.  SPC assessment: Both external teams concluded SPC need—the internal team did not conclude SPC need.  Topics viewed differently among teams and possibly relevant regarding disagreement in SPC assessment:   \| **Learning disability** \| **Support for treatment decision** \| **Discharge management** \| \| --- \| --- \| --- \| \| **Internal team:** Appreciation for the social participation of the patient despite disability; trust in and positive assessment of the support system in place.  **External team 1:** Discussion of (a) the patient’s comprehension of the consequences of a decision against tumour-specific therapy, (b) the sense of radiotherapy in light of the suffering it causes.  **External team 2:** No in-depth discussion. \| All teams agreed on need, but the background for the decision varied:  **Internal team:** Discussion of tumour board recommendation of a future systematic therapy 🡪 offer of one-time expert consultation with sister sufficient (🡪 no SPC).  **External team 1:** Discussion of (a) harm–benefit ratio of radiotherapy, (b) need for advance care planning for emergency situations.  **External team 2:** No in-depth discussion. \| All teams agreed on need. Two teams felt this was a task for SPC services, one did not.  **Internal team:** Confident that the support system in place will manage the situation well:  *D: “The (welfare org.) will take care of it […]*  *A: […] I thought it was fantastic when you told me they came with two people (welfare org.), I thought, wow someone really cares.*  *H: And he will definitely be [a case] for discharge management, […] they have to look for a place for him.”*  **External team 1:** SPC to work out solution with sister.  **External team 2:** SPC to check hospice as possible solution. \|   Most likely reason for disagreement: Direct experience of the patient’s support system by the internal team during medical history taking, which resulted in trust that matters were being taken care of well by the sister and the welfare organisation in cooperation with oncology discharge management—without SPC involvement. |
| --- | --- | --- | --- | --- | --- | --- |

| **Case C - SPC need in patient without current, relevant needs in physical dimension:** Male patient (>40 years, self-employed cook) with adenocarcinoma of the oesophagogastric junction, initially lymphatic metastasised now three cerebral foci. His situation is characterised by no / very low support needs on the physical dimension (occasionally mild chest pain, uncontroversial treatment) but a high need concerning practical problems and psychosocial support (difficult financial situation, inability to continue his job, wife abuses alcohol).  SPC assessment: The internal team and external team 1 concluded no SPC need. External team 2 concluded SPC need with a very close 3:2 vote that was described by the team as *“clearly not clear”*. The case had been included in the 20 selected cases due to the internal team having a similarly difficult time making a decision and being particularly interested in the other teams’ assessments.  Topics viewed differently among teams and possibly relevant regarding disagreement in SPC assessment:   \| **No (current) needs in the physical dimensions, high needs regarding psycho-spiritual and social services support** \| \| --- \| \| All teams concluded similar needs profiles.  **Internal team:** Need of support from psychologist and social worker not sufficient to result in SPC need; expected to be sufficiently addressed in rehabilitation after discharge and by support system in place. Team astonished by the discrepancy between the objective disease situation and the well organised, hardly impaired patient they met.  **External team 1:** No in-depth discussion.  **External team 2:** Discussion of the possibility of a fast progression of the illness resulting in needs in physical dimension and SPC in near future. That led to the some members of the team wishing to monitor the patient’s situation and a discussion of SPC need vs. only offering information on accessibility of specialist palliative care. \|   Most likely reason for disagreement: It was a very narrow decision in external team 2, resulting from the anticipation of support needs in the physical dimension, which was not discussed explicitly by the other teams. |
| --- | --- | --- |
