# Supplementary material for: Interrater agreement of multi-professional case review as reference standard for specialist palliative care need: a mixed-methods study
Source: BMC Palliat Care. 2023 Nov 16;22:181. doi: 10.1186/s12904-023-01281-7 (PMC10652431; doi:10.1186/s12904-023-01281-7)
Supplement: Supplementary file 4 — Additional file 4. Observations on the case review procedure. Observations on the case review process, including effort of case reviewers, framework conditions, supportive needs profile, and oral case presentation. [file 12904_2023_1281_MOESM4_ESM.docx]

**Additional file 4**

**Observations on the case review process**

**Effort of case reviews:**

The average duration of the multi-professional case reviews was very similar in the three teams, with an overall average of m = 9:30 minutes per case (internal team m = 9:36 min, external team 1 m = 9.12 min, external team 2 m = 9.36 min). There were big differences among cases, with some being decided very quickly in a minimum of 4:12 minutes and others taking very long, with up to 23:12 minutes.

The effort and resources required for medical history taking were not recorded.

**Framework conditions**

The guidelines for the case review process were identical for the three teams. However, the prerequisites differed: In Freiburg (internal team), the team discussed ***real world cases*** (e.g. met with patient, could initiate measures)—as opposed to the external teams that relied on case presentations. The Freiburg team often referred to their experiences with the patients and the current support system during the case review and also expressed emotional reactions (e.g. compassion) more often.

Differences in ***team size and composition (professions)*** *as well as* ***experience, knowledge, roles and personalities of team members*** influenced conversation dynamics and content. Examples for possible influences on the needs profile and or SPC need assessment include more frequent questioning of the benefits of current oncologic therapy in one team or different ways of dealing with the expectation of future SPC needs.

**Supportive needs profile**

The use of some ***supportive needs categories*** differed among teams. The assignment of burden to the spiritual and / or psychological needs categories was not always clear. For the Freiburg team, it was also important to distinguish between the two categories of needs ‘treatment continuity’ (continuous support of patient by the same professionals) vs. ‘information, networking and coordination of involved professionals’; however, that was considered an artificial distinction by members of external teams.

**Oral case presentation**

The case descriptions were the same on paper, but the oral presentations differed slightly in some cases.
